# Modification Mechanisms and Properties of Poplar Wood via Grafting with 2-Hydroxyethyl Methacrylate/N,N′-methylenebis(acrylamide) onto Cell Walls

**DOI:** 10.3390/polym15081861

**Published:** 2023-04-13

**Authors:** Jihang Hu, Xiaoqing Wang

**Affiliations:** Research Institute of Wood Industry, Chinese Academy of Forestry, Beijing 100091, China

**Keywords:** wood modification, reaction mechanism, cell walls, grafting, properties

## Abstract

As the only renewable resource among the four basic materials (steel, cement, plastic, wood), wood itself and wood products have a “low carbon” value and play an important role in storing carbon. The moisture absorption and expansion properties of wood limit its application scope and shorten its service life. To enhance the mechanical and physical properties of fast-growing poplars, an eco-friendly modification procedure has been used. This was accomplished by the in situ modification of wood cell walls by vacuum pressure impregnation with a reaction of water-soluble 2-hydroxyethyl methacrylate (HEMA) and N,N’-methylenebis(acrylamide) (MBA). The anti-swelling efficiency of HEMA/MBA-treated wood was improved (up to 61.13%), whereas HEMA/MBA-treated wood presented a lower weight-gain rate (WG) and water-absorption rate (WAR). It was observed that the modulus of elasticity, hardness, density, and other properties of modified wood had improved significantly, as indicated by XRD analysis. Modifiers diffuse primarily within cell walls and cell interstices of wood, causing crosslinks between the modifiers and the cell walls, reducing its hydroxyl content and blocking the channels for water movement, thereby enhancing its physical properties. This result can be obtained by scanning electron microscopy (SEM) and energy dispersive x-ray spectroscopy (EDX), Nitrogen adsorption test imaging ATR-FTIR (Attenuated total reflection-Fourier-Transform Infrared) Spectroscopy, and nuclear magnetic resonance (NMR) and Nitrogen adsorption test. Overall, this straightforward, high-performance modification method is crucial for maximizing wood’s efficiency and the sustainable development of human society.

## 1. Introduction

As a sustainable source, wood has a high carbon-storage and sequestration capacity [1]. Wood has excellent properties and is widely used in furniture, interiors, and structural buildings, playing an important role in the daily lives of people. In order to reduce carbon dioxide emissions, science-based and environmentally friendly methods for processing and utilizing wood can be utilized to maintain carbon stocks in a solid form [2,3]. Plantation wood provides a solid foundation for the wood industry in China. There is no doubt that fast-growing wood is a plentiful and affordable material, but its application in some fields with high requirements for mechanical properties is limited as a result of its common defects, such as easy deformation and cracking as well as a poor dimensional stability [4]. Thus, it is essential to improve wood’s dimensional stability to maximize wood utilization. Furthermore, the development of scientific and ecological applications of plantation wood is vital to the development of a low-carbon economy, as well as to the reduction of the over-exploitation of natural resources.

The cell wall plays an essential role in the macroscopic physical and mechanical properties of wood, which affect the processing and utilization of wood [5]. Consequently, the enhancement effect of wood’s physical and mechanical properties is largely determined by whether modifiers are able to enter cell walls and chemically react with the components of cell walls in order to significantly improve the modification effect and durable stability; thus, it is crucial to study the directional enhancement of wood cell walls [6]. There are many hydroxyl groups in wood cell walls, making them highly reactive. It has been demonstrated that polymer monomers can be very effective in strengthening wood cell walls, improving dimensional stability, reducing water absorption, providing a longer lasting effect, and exhibiting a high resistance [7,8,9]. In particular, the hydrophilic modifiers used in this study were able to better penetrate into the micropores of the cell wall under vacuum pressurization, but further research is needed to clarify the mechanisms involved in the reaction between the modifier and the wood.

Chemical modification was environmentally optimized, since only heat and water-solubility modifiers were used in this study. There are -OH groups in 2-hydroxyethyl methacrylate (HEMA), which is a non-toxic, biocompatible, water-soluble monomer that is readily polymerized and copolymerized as well as capable of forming three-dimensional interconnected network polymers when cross-linking agents are applied [10]. Generally, HEMA-based copolymers exhibit a good hemocompatibility and biocompatibility, are non-toxic, and show good prospects for medical applications [11]. These characteristics make it an ideal material for contact lenses, drug release, the production of artificial leather, and tissue engineering, among other applications [12,13]. According to studies, the water absorption of wood modified with hydroxyethyl methacrylate (HEMA) was reduced, and the hardness and dimensional stability were improved [14,15]. However, different crosslinkers or initiators can affect the reaction between HEMA and wood, which can ultimately affect the performance of the modified wood, so further research on HEMA’s use on wood is required. Typically used to prepare high-molecular gel materials, MBA has excellent reactivity properties and biocompatibility [16,17,18]. Because of the existence of vinyl, an ester network is formed when water-solubility 2, N-methylenebisacrylamide (MBA) and 2-hydroxymethacrylate (HEMA) are reacted with wood cell walls using initiators. A stable reticular structure may be formed when HEMA and MBA interact with the components of wood, resulting in modified wood with a good dimensional stability. In order to achieve the enhanced effect, the modifier must be cured under certain conditions and chemically combined with the component of the cell wall.

Low weight-gain rates were achieved by heat-inducing 2-hydroxyethyl methacrylate and N,N-methylenebisacrylamide (MBA) to polymerize in situ within the cell wall. In this research, hydrophilic hydroxyl groups in wood cell walls are grafted by HEMA/MBA to enhance dimensional stability and water absorption. The present study aims to improve the properties of wood by synthesizing macromolecules with regulated structures. The in-depth analysis of the mechanism underlying the reaction that occurs between wood cell wall components and modifiers will contribute to the development of a potential method for wood modification. 

## 2. Experimental Materials and Methods

### 2.1. Experimental Materials

Poplar wood (*Populus euramevicana* cv.I-214) was gathered at the Sun jiazhuang forest farm in Yi County, Hebei province, China. The trees were collected at 15 years of age with an average height and diameter at breast height of 18.16 m and 23.76 cm. Without defect faults and sawed to boards, the poplars were sawed into 20 mm × 20 mm × 20 mm, to be absolutely dry so as to be prepared for the impregnation experiment. Beijing Yixiubogu Chemical Products Co., Ltd. (Beijing, China) supplied HEMA. MBA-99.0%, chemical grade, was purchased from Aladdin Co., Ltd. (Shanghai, China).

### 2.2. Wood Modification Methods

The aqueous solution modifier with different HEMA-to-MBA mass ratios was prepared for following impregnation. Moreover, H_2_O_2_ and CaCl_2_ were used as initiators. According to Table 1, different impregnation solutions have different ratios.

Through vacuum pressure, the above-prepared solution was impregnated into wood samples in the following order: vacuuming (0.1 MPa, 1 h), liquid injection, pressurization (1.0 MPa, 8 h), and pressure relief. Following pressure release and air-drying for three days, the samples were heated to 40 °C for 24 h followed by 80 °C for 12 h in order to initiate the reaction between the modifier and the wood cell wall. All samples were dried at 103 ± 2 °C for a constant weight.

### 2.3. Characterization

#### 2.3.1. Scanning Electron Microscope (SEM) and Energy-Dispersive X-ray Spectroscopy (EDX)

At 10 kV, SEM analysis (Hitachi SU8020, Tokyo, Japan) was used to observe the modifier distribution in wood cell walls and wood rays. Platinum was coated on modified and unmodified wood samples (5 mm × 5 mm × 5 mm) held on conductive adhesives. Furthermore, both modified and unmodified wood samples were assayed by EDX (INCA Energy 300 system, Oxford Instruments, UK) for the characteristic elemental content.

#### 2.3.2. Imaging Fourier Transform Infrared Spectroscopy (FTIR) Microscopy

FTIR imaging measurements were conducted on wood samples sliced into 10 mm transverse slices. An Imaging System (PerkinElmer Inc., Shelton, CT, USA) was operated in Attenuated Total Reflection (ATR) mode. With ATR mode, an area of interest of 100 × 100 mm was scanned randomly in the cross-section of the cell wall. To obtain an IR full-spectrum image of the sample, 16 scans were taken at a resolution of 4 cm^−1^.

#### 2.3.3. Nuclear Magnetic Resonance Spectroscopy (NMR)

NMR (Bruker-AVIII-400 MHz, Aachen, Germany) was conducted on 160-mesh unmodified flour and modified wood flour, stirring DMSO-d6 evenly after adding 30 mg sample. In order to acquire ^13^C NMR spectra of modified wood samples, cross-polarization (CP) and magic angle spinning (MAS) methods were used at room temperature.

#### 2.3.4. X-ray Diffraction (XRD) Analysis

The preset parameters of XRD analysis (Germany-based Bruker D8 Advance diffractograms) were 40 kV, 40 mA, and 2θ scan range from 5° to 45° with a scanning speed of 2°/min. According to the following formula, the crystallinity index (CrI) is calculated:

(1) $$\mathrm{CrI}\;(\%)=\frac{100(I_{002}-I_{am})}{I_{002}}$$

As the amorphous background scattering intensity of *I_am_* is about 18°, *I*_002_ is the crystallographic scattering intensity in the (002) crystallographic plane.

#### 2.3.5. Nitrogen Adsorption Test

Utilizing a nitrogen adsorption device (Autosorb iQ, Quantachrome, Atlanta, GA, USA), samples were analyzed for surface area and pore size distribution at 77 K. The total pore volume (Vtotal) was calculated from the volume of liquid nitrogen maintained at a relative pressure of 0.99. The sample surface area (SBET) was determined by Brunauer–Emmett–Teller (BET). Based on Barrett–Joyner–Halenda (BJH) calculations, the pore size distribution was determined. The nitrogen adsorbed by each group is the mean of three tests.

### 2.4. Wood Performance Assessment

Samples were measured at 20 mm × 20 mm × 20 mm (length × radial × tangential) to determine the weight percentage gain (WPG), bulking effect (BE), anti-swelling efficiency (ASE), and water absorption (WAR). A statistical average was determined for each group based on ten replicates.

WPG was determined by the following Formula (2):

(2) $$\mathrm{WPG}\;(\%)=\frac{100(W_{1}-W_{0})}{W_{0}}$$

Here, *W*_0_ and *W*_1_ represent the weights of oven-dried wood samples prior to and following treatment.

A BE test (Formula (3)) is used to evaluate the dimensional changes in wood after and before modification.

(3) $$\mathrm{BE}\;(\%)=\frac{100(V_{1}-V_{0})}{V_{0}}$$

Wood before and after impregnation has different drying volumes, *V*_0_ and *V*_1_, respectively.

ASE was calculated by measuring the volumes of modified and unmodified samples before and after immersion in water for 10 days.

(4) $$\mathrm{VSE}\;(\%)=\frac{100(V_{a}-V_{b})}{V_{b}}$$

The wood sample’s volume before and after immersion is represented by *V_a_* and *V_b_* here.

(5) $$\mathrm{ASE}\;(\%)=\frac{100{(VSE}_{u}-{VSE}_{t})}{{VSE}_{u}}$$

The efficiency of the volume expansion of unmodified and modified wood is expressed by *VSE_u_* and *VSE_t_*.

The water absorption rate (WAR) was calculated according to GB/T 1934.1-2009 by recording the weight of the modified wood before soaking and after 10 days in water.

(6) $$\mathrm{WAR}\;(\%)=\frac{100(W_{2}-W_{1})}{W_{1}}$$

As shown here, *W*_1_ and *W*_2_ represent the mass of wood after modification and after soaking, respectively.

According to GB/T 1933-2009, GB/T 1941-2009, and GB/T 1936–2009, we measured the modulus of rupture (MOR) and modulus of elasticity (MOE), as well as the density and hardness.

## 3. Modification Mechanisms and Performance Characterization

### 3.1. Modifier Distribution Analysis

**Figure 1:**
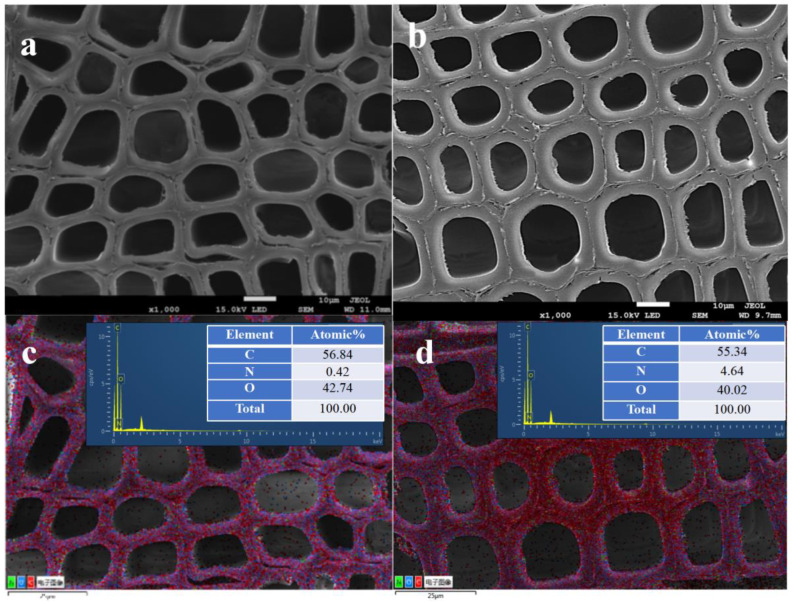
The morphological characteristics of unmodified wood compared to modified wood with HEMA-to-MBA mass ratios of 40 wt % and 2 wt %. (**a**) and (**b**) are SEM images from unmodified and modified wood, respectively. (**c**) and (**d**) represent the energy spectra of unmodified and modified wood, respectively.

**Figure 2:**
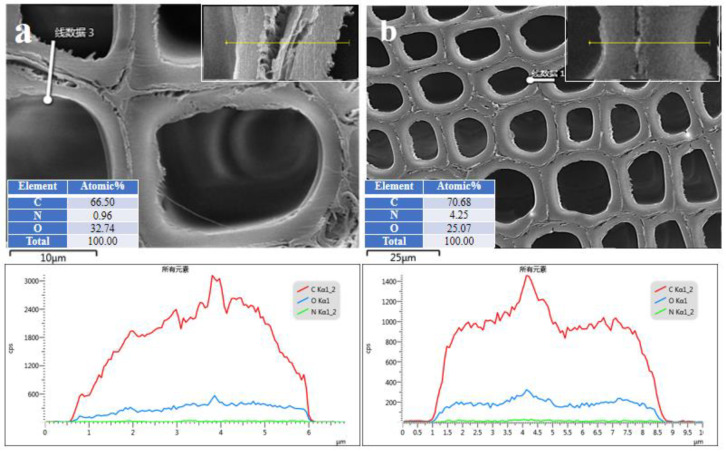
The morphological characteristics of unmodified wood compared to modified wood with HEMA-to-MBA mass ratios of 40 wt % and 2 wt %. Unmodified and modified woods are scanned linearly, respectively, in (**a**) and (**b**).

Unmodified wood has thinner cell walls than modified wood, as shown in Figure 1a,b; additionally, there are visible fractures between the cell walls in unmodified wood, but almost no gaps between the cell walls of the modified material. Through the addition of modifier monomers, polymers are formed that help fill the cell walls. Accordingly, the performance of the modified wood was improved by strengthening the cell wall with the modifiers. The energy spectrum analysis shows that oxygen, carbon and nitrogen were distributed primarily in the cell wall, while trace amounts were distributed in the cell lumen, indicating that the modifying agent penetrated the cell wall. Energy Spectrum Analysis showed a pre-treatment nitrogen content of 0.42% and a post-treatment nitrogen content of 4.64% (Figure 1c,d). It can be seen from the linear scanning of the energy spectrum figure (Figure 2) that the relative content of the nitrogen is increased in cell walls and cell gaps after modification. This indicates that the modifier is mainly located in the cell walls and gaps. WPG and BE are important indicators to evaluate the penetration of modifiers in wood. The maximum weight gain of modified wood can reach 18.86% (±2.47), and the capacity gain reached 11.2% (±1.03), indicating that the modified wood can not only penetrate the wood but also the cell wall.

### 3.2. Modification Reaction Mechanism

**Figure 3:**
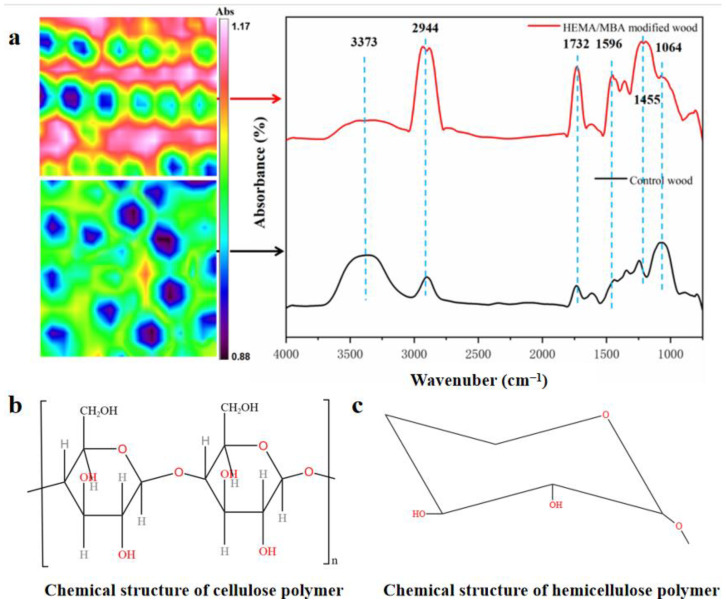
*(***a**) The FTIR spectrum of the modified and unmodified wood. (**b**,**c**) are the chemical structures of cellulose and hemicellulose, respectively [22,23].

**Figure 4:**
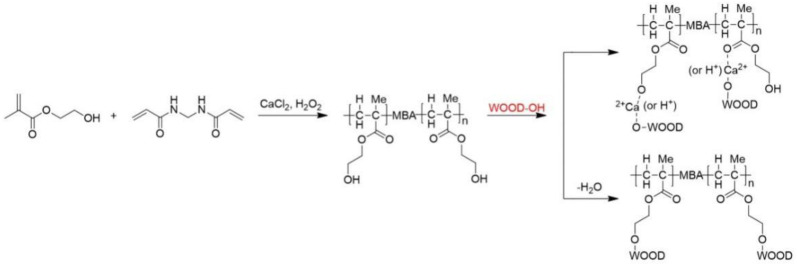
The reaction formula of HEMA and MBA with wood.

Figure 3 illustrates HEMA and MBA adsorbed on modified and unmodified woods. HEMA and MBA grafts produced weaker absorption peaks near 3373 cm^−1^, which suggests that -OH on wood played a role in grafting [19]. A strong C-H telescopic vibration and bending vibration wind appeared in 2944 cm^−1^ and 1455 cm^−1^, mainly due to the alkanes of the modifier. It should be noted that the two prominent peaks (1732 cm^−1^ and 1596 cm^−1^) observed in the spectrum are due to vibrations of –C=O stretching (Amide I) and –NH bending (Amide II), respectively (Figure 4). Additionally, cellulose or hemicellulose showed a weakening in the peak of the C-O stretching vibration at 1064 cm^−1^, indicating a reaction of esterification between the modifier and the cellulose or hemicellulose [20,21].

**Figure 5:**
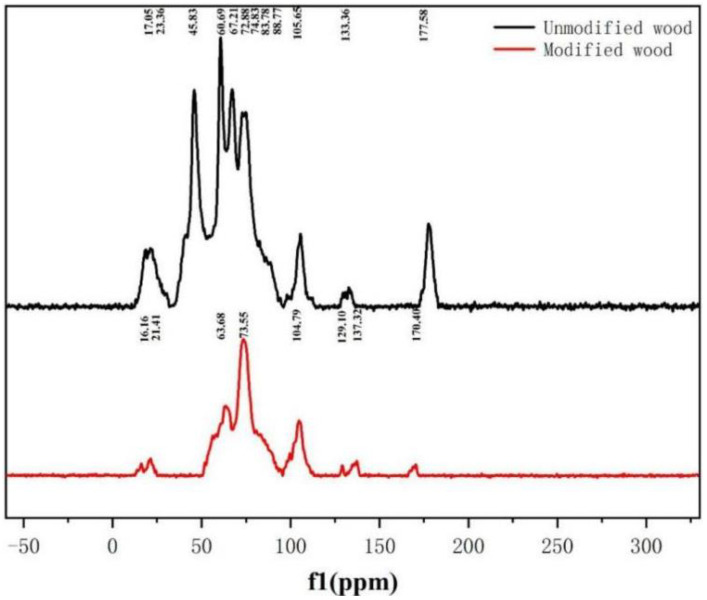
NMR spectroscopy of unmodified and modified wood.

NMR was performed to examine whether the modified wood showed a new chemical shift after interacting with HEMA/MBA and whether a chemical reaction occurred in the modified wood, in order to confirm the finding of the FTIR. The ^13^C NMR spectrum of treated and untreated poplar wood is shown in Figure 5. The most obvious signal peak is mainly concentrated in 50–110 ppm, which represents the C6 of cellulose. C6 is represented by 67 and 63 ppm in the crystalline and amorphous regions. The signal peaks of C2, C3 and C5 mainly appear at 73 and 75 ppm [24,25]. The curve in Figure 5 shows that the main characteristic peak of HEMA is a reaction with wood at 133.36, 88.77, 83.78, and 74.8 ppm. The characteristic peaks at 133.36 ppm belong to the structure of R-CH = C and R-C = C, mainly due to the introduction of C = O [26]. The characteristic peaks of 83.78 ppm and 88.77 ppm indicate that the crystalline structure of cellulose is not damaged, but an etherification reaction occurs in the amorphous region of cellulose, leading to -OH decreasing in the cellulose [27]. Figure 5 shows that the intensity of the peaks at 83.78 and 88.77 ppm is relatively weak, which is also a manifestation of the decrease of hydroxyl. The NMR of carbon shows that the hemicellulose peaks are located at 23.36, 60.69, 74.83, 105.65 and 177 ppm, representing, respectively, C6 in methoxy, C2–C5 in polysaccharides, C4 in xylan, Cl in hemicellulose, and carbonyl carbon in the acetyl group [28]. According to Figure 5, except for the peak at 21 ppm, the peak intensity of other peaks decreased. This is primarily the result of the unstable structure of hemicellulose being degraded during treatment.

**Figure 6:**
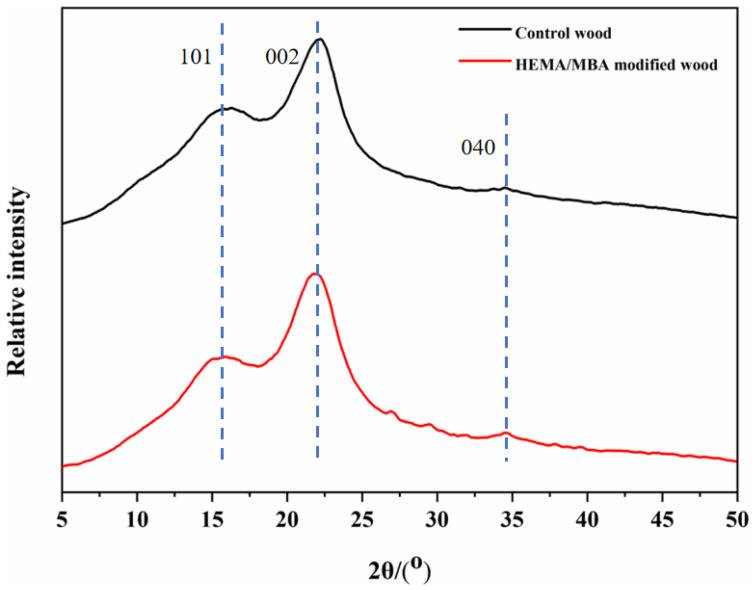
XRD of modified and unmodified wood.

Wood properties are affected by crystallinity, which has an impact on hardness, the modulus of elasticity, density, and dimensional stability [29]. Diffraction peaks in control wood are at 2θ = 16.1°, 22.2°, 35°, corresponding to the (101), (002), and (004) crystallographic planes of cellulose, respectively. According to Figure 6, the intensity, not the position, of the diffraction peaks changed after modification. Compared to the unmodified wood (37.4%), the wood modified by HEMA/MBA had a significantly higher relative crystallinity (48.1%). Modifiers cause the cellulose non-crystalline region of the cell wall to have a more orderly structure, increasing the relative crystallinity in the wood, which ultimately enhances its mechanical properties and dimensional stability. Modified wood displays an increase in dimensional stability and absolute dry density as crystallinity increases, which is consistent with the testing results of its properties.

### 3.3. Moisture Sorption and Dimensional Changes

**Figure 7:**
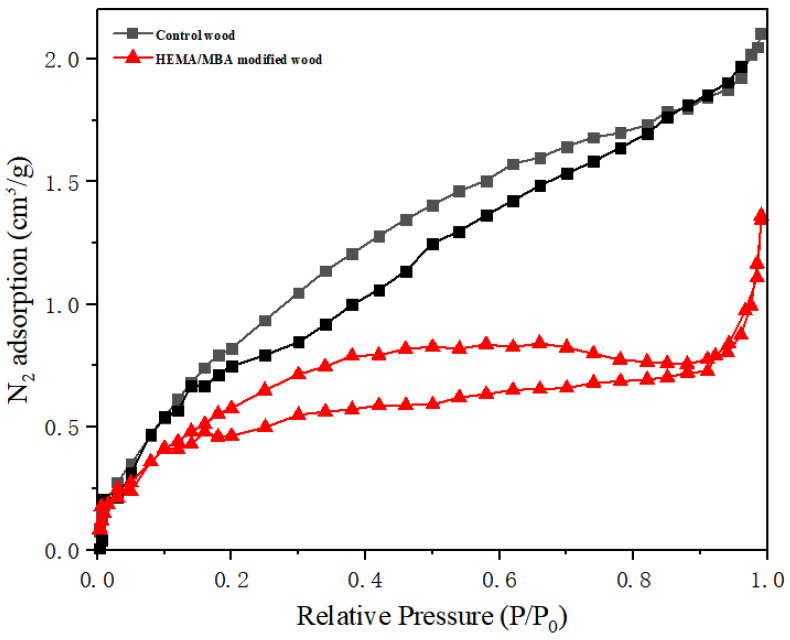
Nitrogen adsorption-desorption isotherms.

Water molecules diffuse through wood based on the size and distribution of pores [30]. Nitrogen adsorption was applied to characterize the modification influence of HEMA/MBA on wood pore structure. Nitrogen adsorption and desorption isotherms for unmodified and modified wood are shown in Figure 7, showing that the amount of nitrogen adsorption was significantly reduced after the wood was modified with HEMA/MBA. This may be caused by the blocking of some pore polymers in the pores of the wood. In addition, Table 2 shows a reduction in the total pore volume and specific surface of the modified wood, while the total pore volume decreased by 48.5%, suggesting that the polymer formed by the monomer blocked some of the pores in the wood, thus reducing its porosity.

### 3.4. Dimensional Stability and Mechanical Characteristics

#### 3.4.1. Effect of HEMA Dosage on Properties of Modified Wood

**Figure 8:**
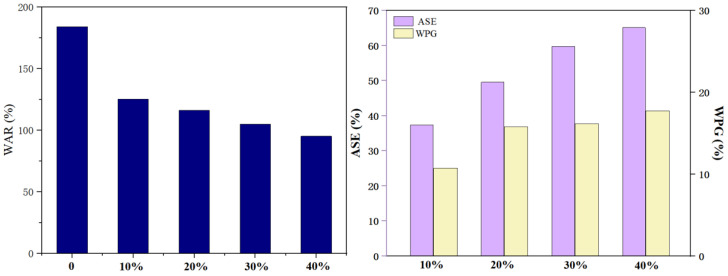
Effect of HEMA dosage on wood properties.

By interacting with wood cell walls, the modifiers can improve the performance of wood cell walls. MBA is a reactive crosslinker, while HEMA is a bifunctional crosslinker. HEMA and MBA can form polymers through vinyl and form three-dimensional structures through hydroxyl cross-linking reactions to reinforce the cohesiveness of polymers and improve the physical performance of composites [31]. Therefore, the dosage of HEMA and MBA directly affects the modification effect of wood. As shown in Figure 6, HEMA dosage has a positive effect on the ASE, WPG, and WAR of wood, while other factors are maintained constant (MBA addition was 2%). It can be observed from Figure 8 that the WPG of modified wood increased positively with HEMA concentrations. According to this trend, the higher the modifier concentration, the more modifiers remain inside the wood cell walls. Pressure impregnation allows the modifiers to effectively penetrate and fix into the wood. Moreover, modified wood becomes more dimensionally stable as the modifier concentration increases. The weight-gain rate of the modified wood is related to the dimensional stability. When the weight-gain rate was 10.73% and 17.85%, ASE reached 37.40% and 61.13%. By polymerizing in the wood, the modifiers fill the pores in the wood cell walls, preventing water from entering the wood and improving its dimensional stability.

Figure 8 shows how the WAR varies with the modifiers’ concentration. A significant reduction in the WAR of modified wood can be achieved through modification, and the WAR declines with an increase in the modifier concentration. Modified wood had the lowest WAR at a 40% HEMA concentration, 95.58%. Furthermore, the modifiers prevented water from flowing through conduits, pipe holes, and grain holes in the wood, resulting in a reduction in WAR.

#### 3.4.2. Effect of MBA Dosage on Properties of Modified Wood

**Figure 9:**
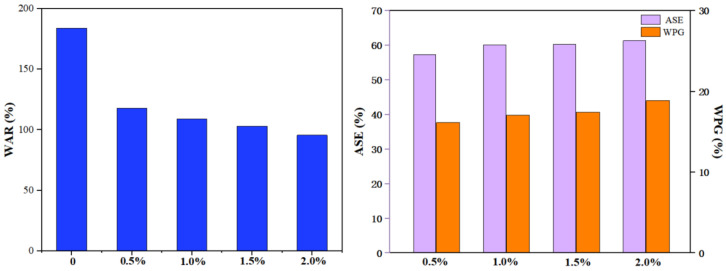
Effect of MBA dosage on wood properties.

Figure 9 reflects that the dosage of MBA has an influence on ASE, WPG and WAR, while other factors are maintained constant (the HEMA addition was 40%). As shown in Figure 9, with an increasing MBA, the WPG of the modified wood was 16.15%~18.86%. Accordingly, MBA does not significantly affect the WPG of modified wood. Figure 9 suggests an increasing trend in the dimensional stability of modified wood with rising MBA dosages. When the MBA content was 0.5% and 2%, the ASE of modified wood was 57.35% and 61.13%. Modified wood with an increased MBA content had a slightly higher ASE. Through the action of an initiator, vinyl monomers reduce the amount of hydroxyl groups in wood and reduce its WAR by grafting them with hydroxyl groups.

Figure 9 shows the variation in WAR of modified wood with MBA. With a higher MBA addition, the WAR of modified wood decreased significantly compared to the control group. Fixing the modifiers in the wood prevents water from entering and leaving, thus reducing the WAR of wood. A modified material with 40% HEMA and 2% MBA contained the lowest water absorption rate, which was 95%. Interactions between MBA and HEMA increased the cross-linking between wood and modifiers, restricting water fluidity.

### 3.5. Evaluation of Physical and Mechanical Properties

An analysis of the elastic modulus, hardness, and other properties was conducted on samples with optimal processing conditions for dimensional stability (Table 3). As compared to the unmodified wood, the wood modified with HEMA/MBA demonstrated a significant improvement in both physical and mechanical properties, increasing MOE and MOR by 64.1% and 45.0%, respectively, and the absolute dry density by 19.5%. Furthermore, the diameter surface hardness accounted for 29.8%, the chord surface hardness accounted for 47.8%, and the end surface hardness accounted for 45.7%. In accordance with the XRD results, all wood performances were enhanced. According to the SEM and Image ATR-FTIR data, the modifying agents primarily affect the wood cell walls, with a BE of 11.23%.

## 4. Conclusions

Here, a water-soluble vinyl monomer was polymerized in situ with water to enhance the wood’s dimensional stability. Based on the method of etherification reaction by injecting modifier into wood, a cross-linking network system was constructed to promote the wood’s dimensional stability. A water-soluble monomer and crosslinking agent are injected into the wood cell walls, and crosslinking reactions are generated, forming an interpenetrating polymer network system. By partially blocking wood pores, partially changing hydroxyl, and increasing the transverse connection of wood at the same time, one can enhance the wood’s dimension stability significantly. According to the results, the modified wood exhibits a low water absorption, low weight gain, and dimensional stability of up to 61.13%. In addition, both the mechanical and physical performances of the wood were enhanced, confirming that the relative crystallinity of the wood improved.

## Figures and Tables

**Table 1:** Parameters of treatments for each group of wood samples.

The Dosage of HEMA	The Dosage of MBA
10%	2.0%
20%	
30%	
40%	
40%	0.5%
	1.0%
	1.5%
	2.0%

**Table 2:** Pore structure parameters of modified and unmodified wood.

The Dosage of HEMA	S_BET_ (m^2^/g)	V_total_ (×10^−3^ cm^2^/g)
Untreated wood	4.155	1.334
Modified wood	2.177	0.667

**Table 3:** Comparison of modified and unmodified poplars in terms of their physical and mechanical properties.

Group	MOE (Gpa)	MOR (Mpa)	Oven-Dried Density (g/cm^3^)	BE (%)	Hardness (N)	
Unmodified wood	7.8	71.3	0.41	—	Radial load	2580
					Tangential section	2420
					Cross-section load	4070
Modified wood	12.8	103.4	0.49	11.23	Radial load	3350
					Tangential section	4020
					Cross-section load	5930

## Data Availability

All the data of this is included in the manuscript.
